# A rare diagnostic imaging and clinical implications of expansile lytic lesion in the distal femur of a 41-year-old female

**DOI:** 10.11604/pamj.2025.50.4.46027

**Published:** 2025-01-02

**Authors:** Pradhyum Dilip Kolhe, Pratik Phansopkar

**Affiliations:** 1 Department of Musculoskeletal Physiotherapy, Ravi Nair Physiotherapy College, Datta Meghe Institute of Higher Education and Research (DU) Sawangi Meghe, Maharashtra, India,; 2 Department of Musculoskeletal Physiotherapy, School of Physiotherapy, Bharati Vidyapeeth (Deemed to be University) Medical College, and Hospital, Sangli, Maharashtra, India

**Keywords:** Giant bone tumour, oncology, lytic lesion

## Image in medicine

An expansile lytic lesion is a type of bone lesion that may arise from numerous benign or malignant conditions, leading to the destruction of bone tissue. A 41-year-old female presented with a four-month history of progressive left knee pain and intermittent swelling, that worsened by physical activity. There was no history of trauma or fever. On physical examination, grade 3 tenderness over the distal femur with diffuse soft tissue swelling was noted. Anterior-posterior (A) and lateral (B) radiographs revealed a well-defined expansile lytic lesion with cortical thinning and marked expansion in the distal femoral metaphysis. Laboratory investigation, including inflammatory markers and serum calcium level, were slightly raised. Needle biopsy revealed multinucleated giant cells in a background of mononuclear stromal cells which was diagnosed as osteosarcoma lytic variant with expansile lytic lesion in the distal femur. The patient was referred for physiotherapy. Following physical assessment considerate muscle wasting of the left quadriceps and hamstrings, with painful and limited hip and knee movements was noted. The patient underwent physiotherapy rehabilitation of strengthening of hip and knee musculature and gait training with quadrapod stick followed by cryotherapy ice pack for pain management and alleviating swelling. Post rehabilitation of four weeks, the patient reported improved lower extremity functions and reduced pain during activities of daily living.

**Figure 1:**
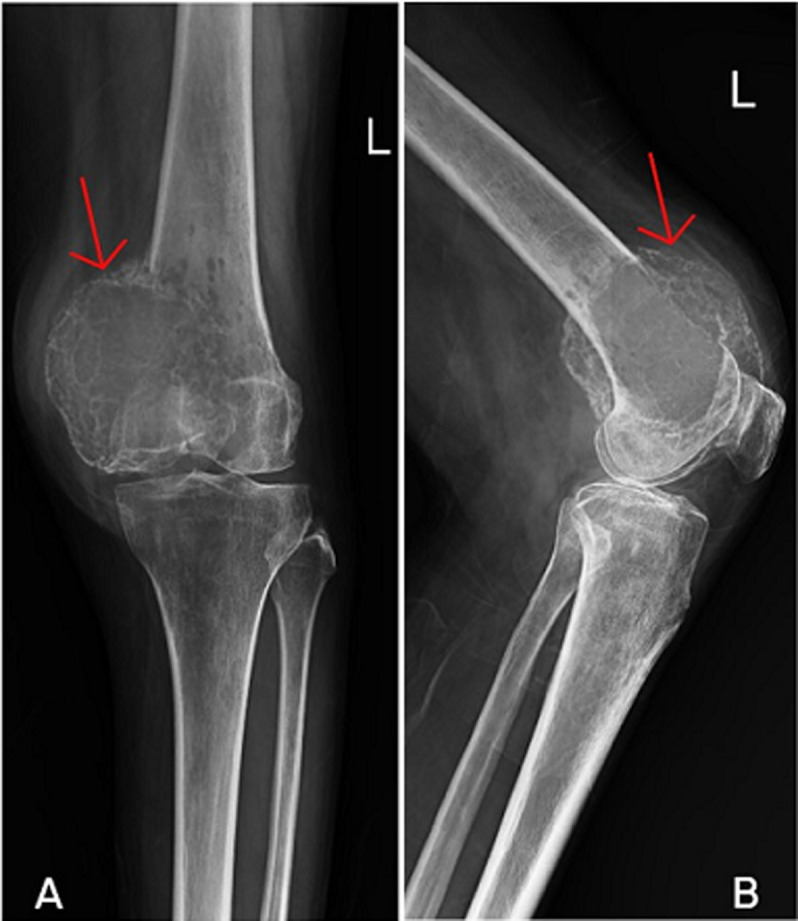
A, B) X-ray showing expansile lytic lesion at the distal femur

